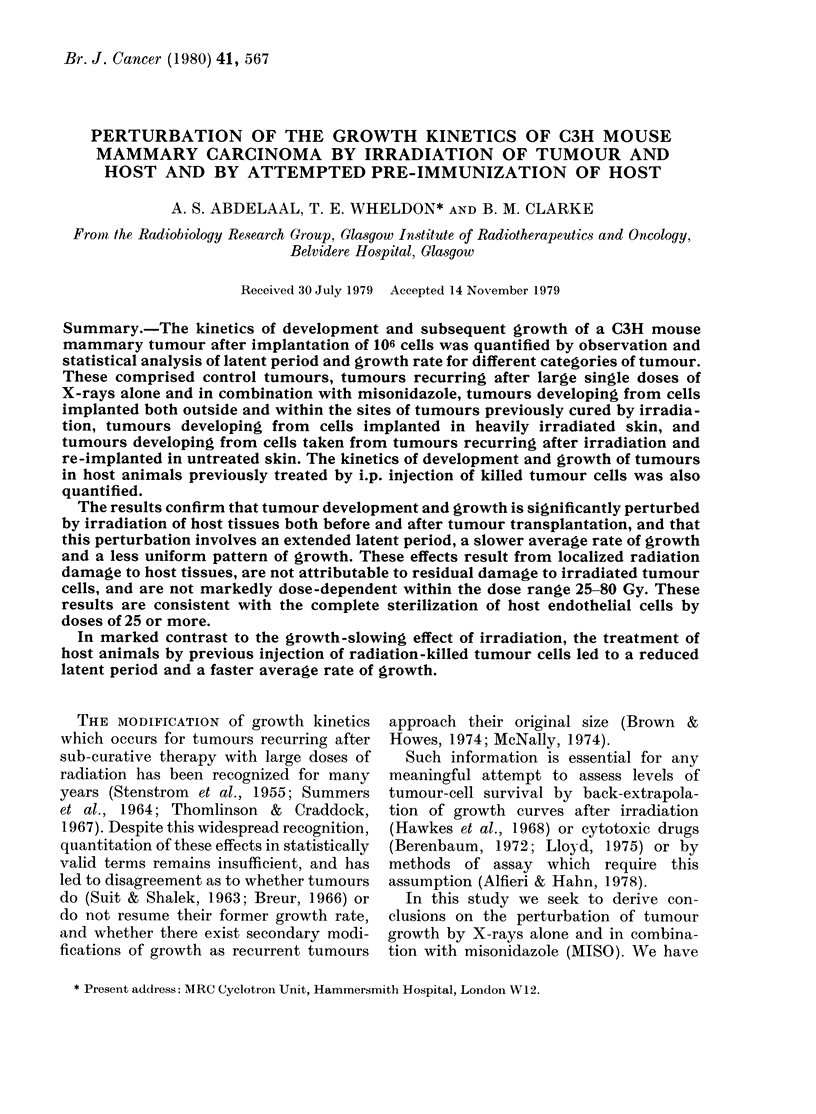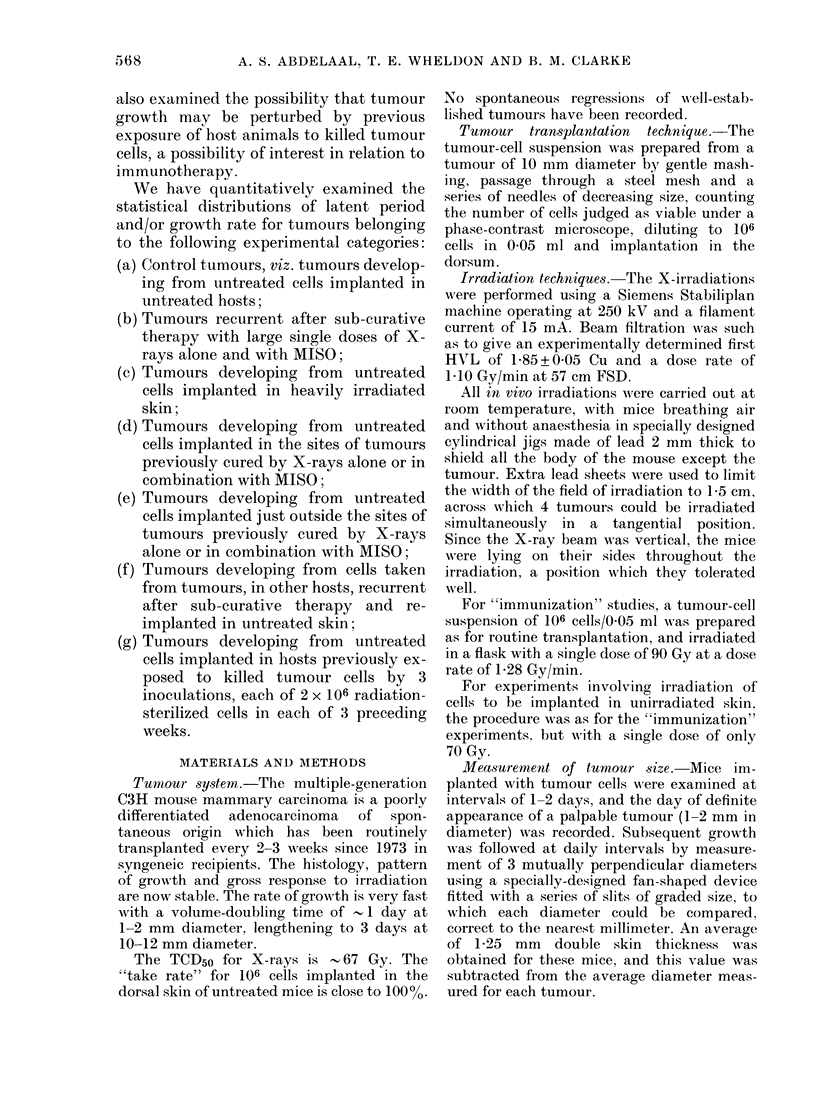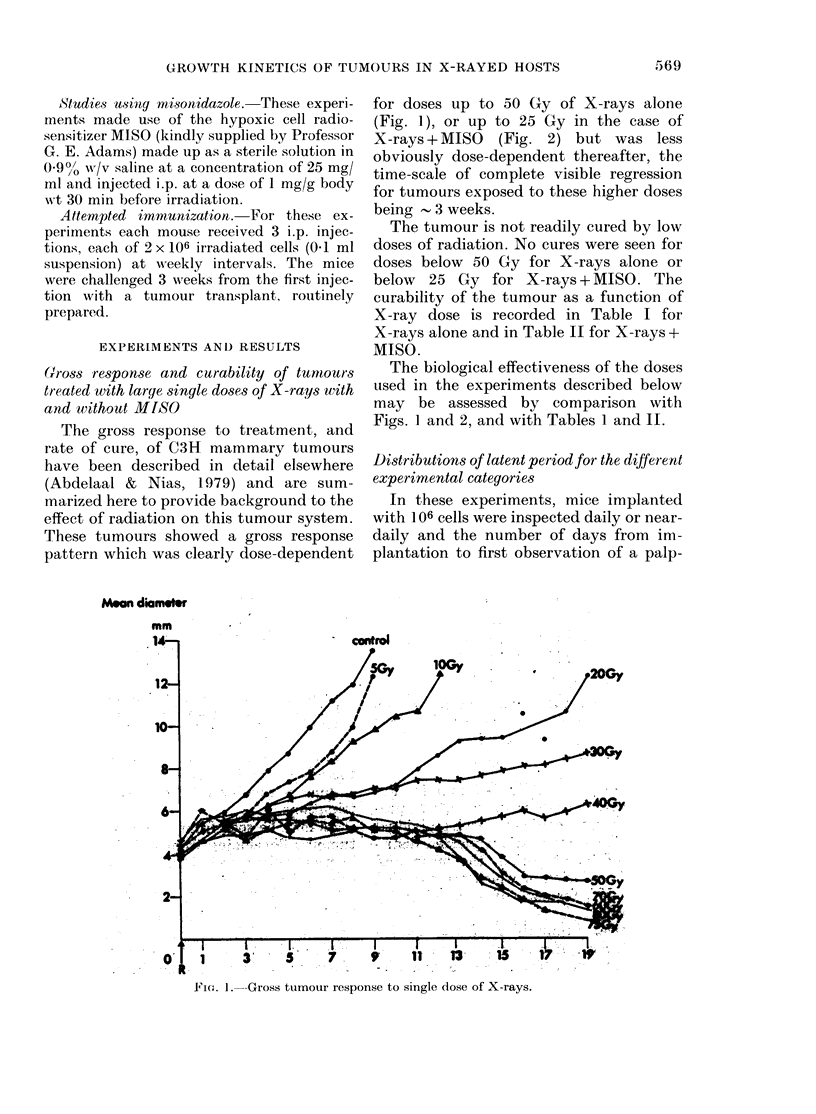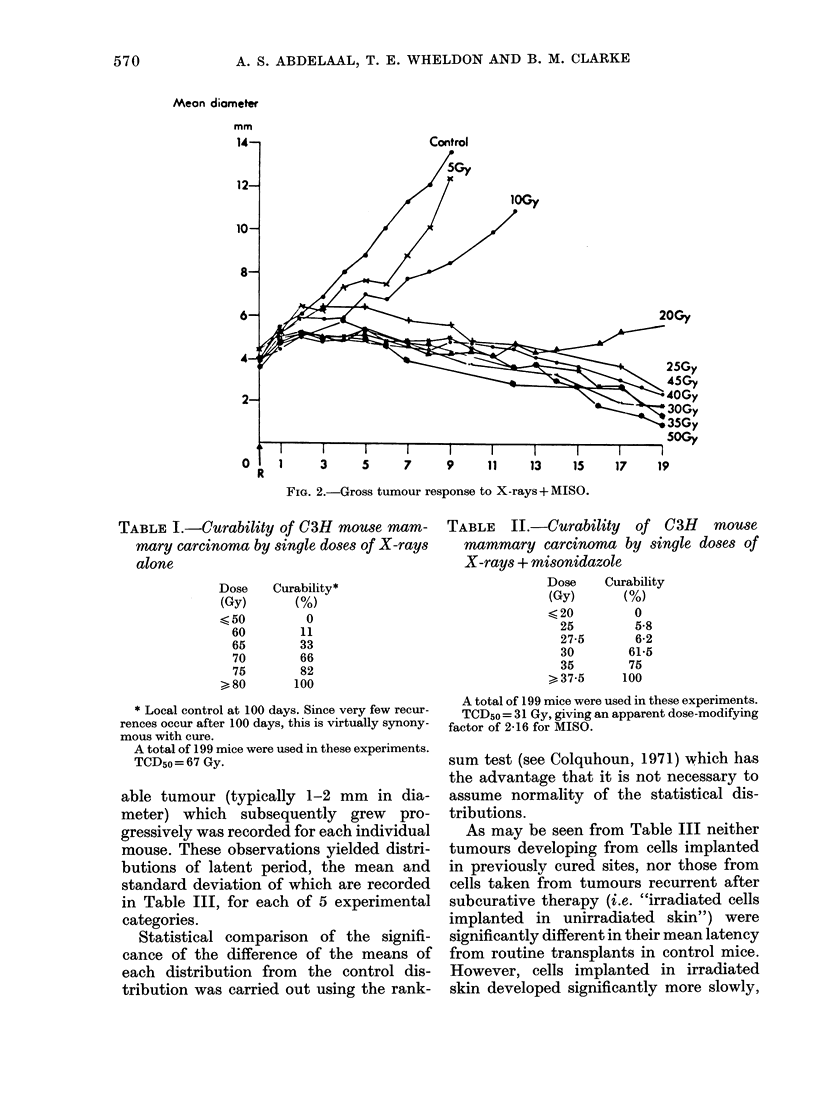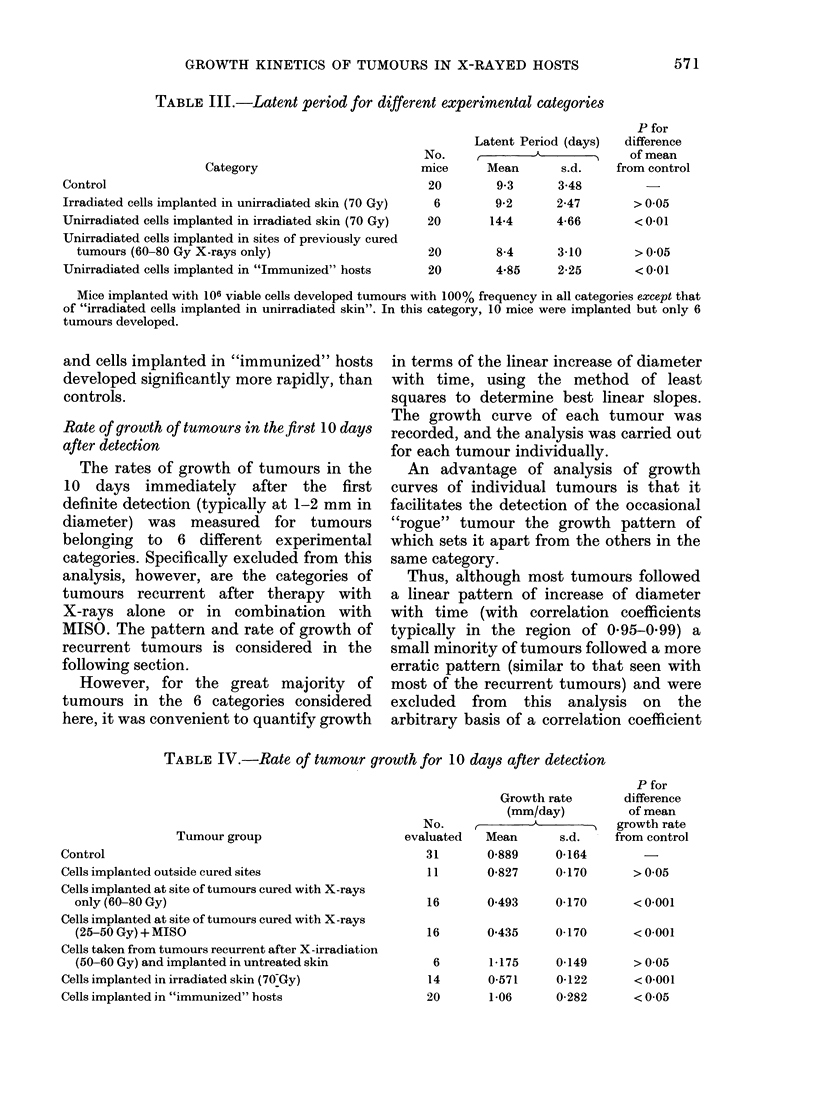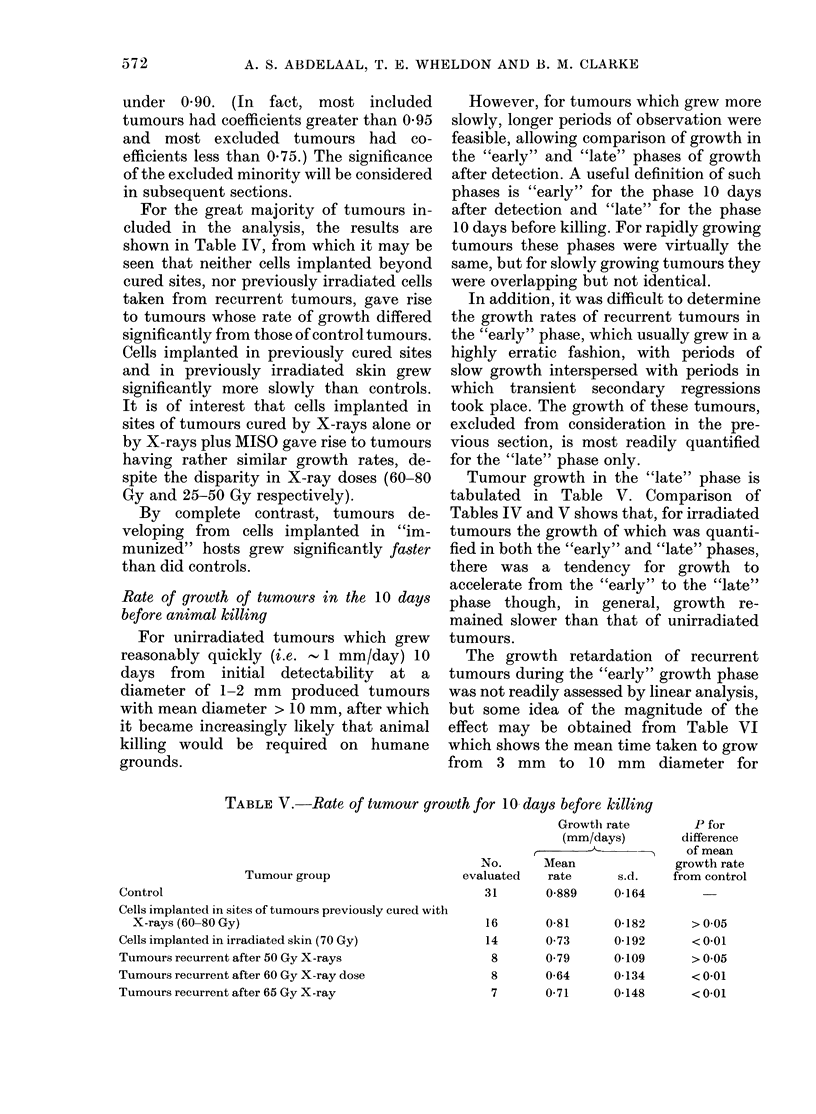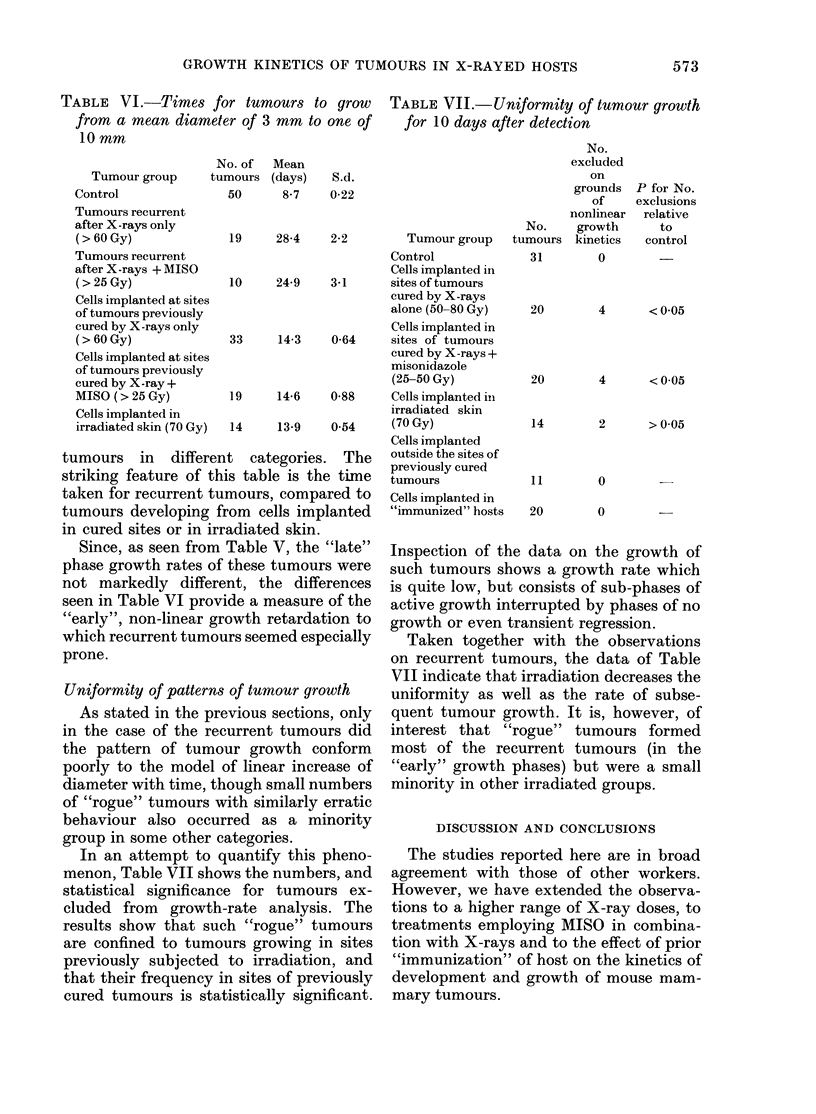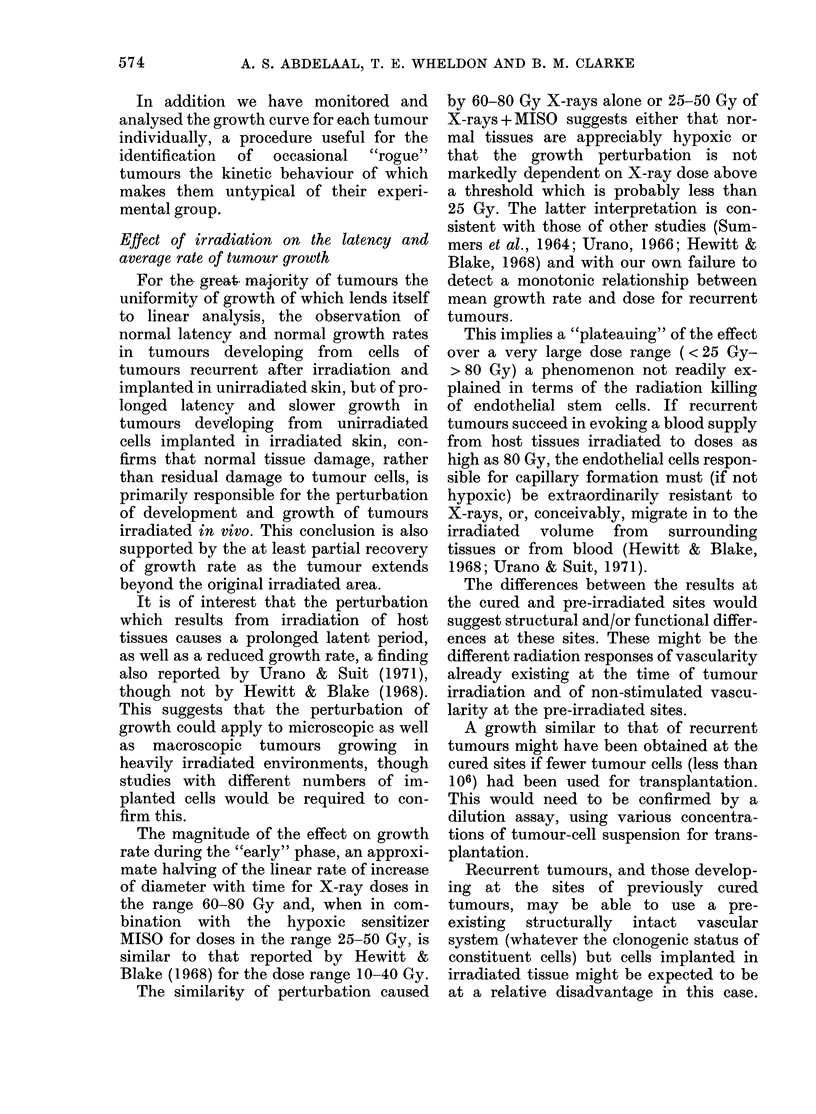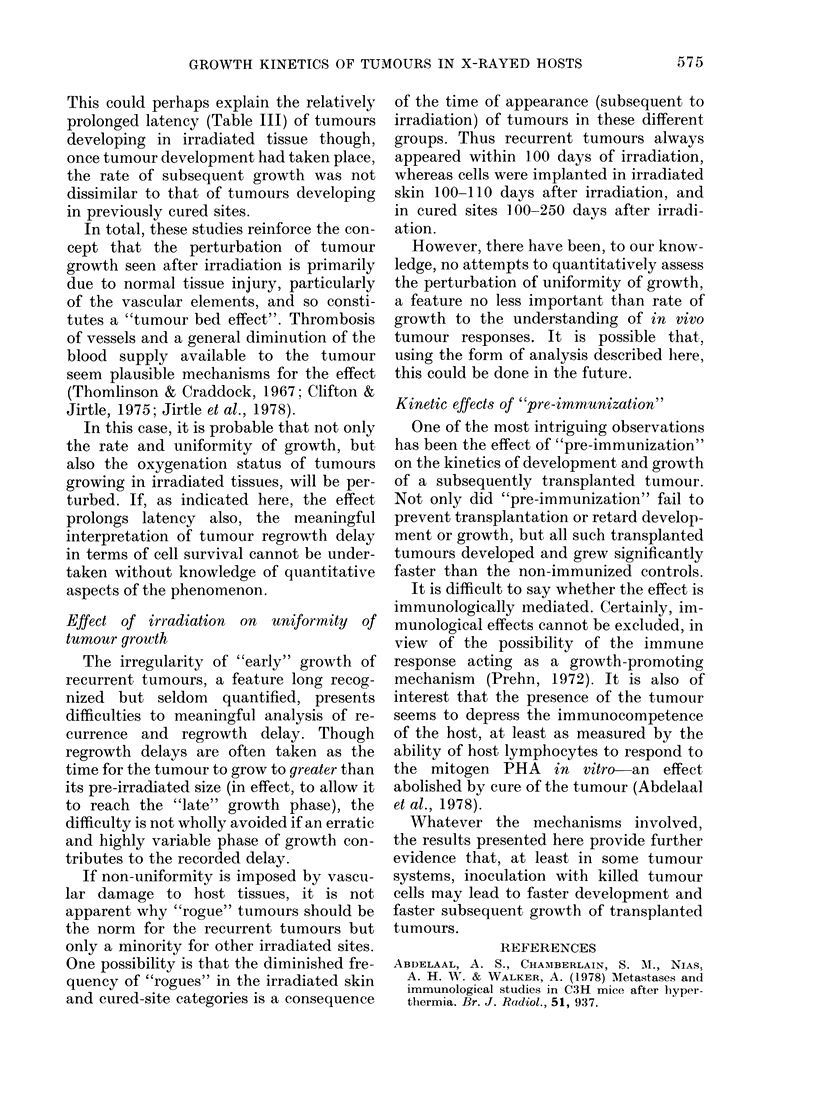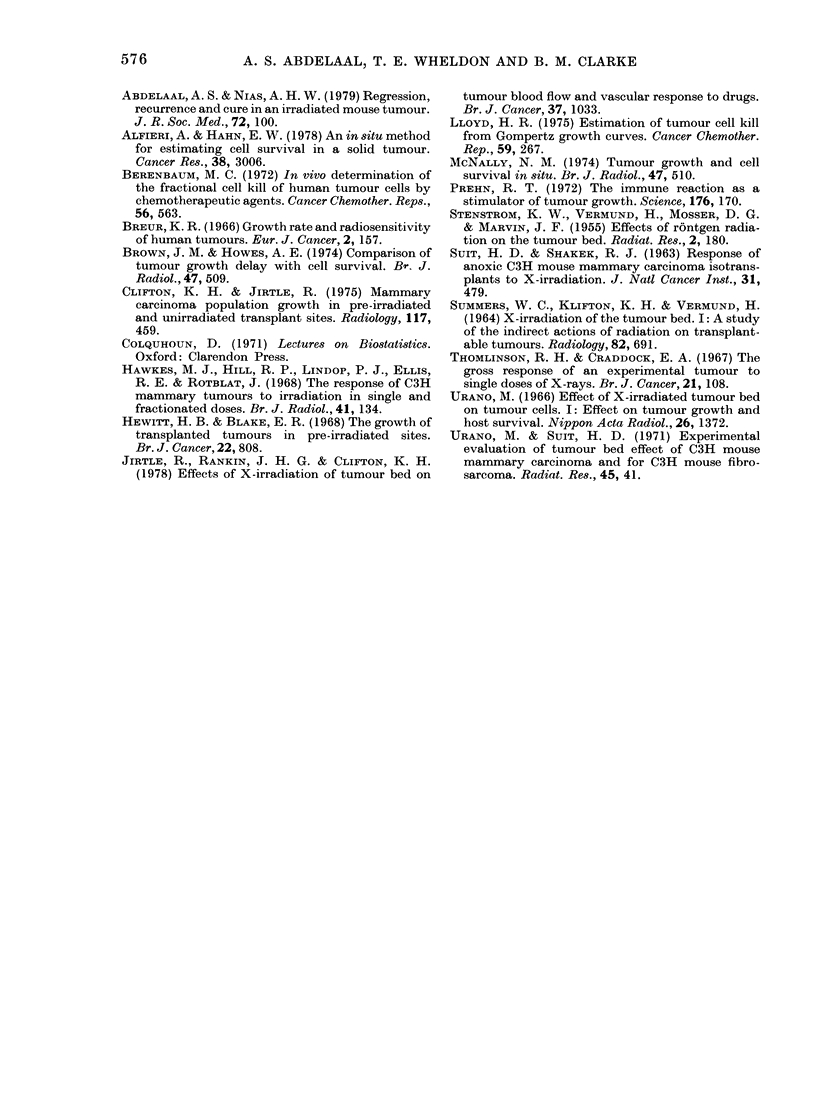# Perturbation of the growth kinetics of C3H mouse mammary carcinoma by irradiation of tumour and host and by attempted pre-immunization of host.

**DOI:** 10.1038/bjc.1980.99

**Published:** 1980-04

**Authors:** A. S. Abdelaal, T. E. Wheldon, B. M. Clarke

## Abstract

The kinetics of development and subsequent growth of a C3H mouse mammary tumour after implantation of 10(6) cells was quantified by observation and statistical analysis of latent period and growth rate for different categories of tumour. These comprised control tumours, tumours recurring after large single doses of X-rays alone and in combination with misonidazole, tumours developing from cells implanted both outside and within the sites of tumours previously cured by irradiation, tumours developing from cells implanted in heavily irradiated skin, and tumours developing from cells taken from tumours recurring after irradiation and re-implanted in untreated skin. The kinetics of development and growth of tumours in host animals previously treated by i.p. injection of killed tumour cells was also quantified. The results confirm that tumour development and growth is significantly perturbed by irradiation of host tissues both before and after tumour transplantation, and that this perturbation involves an extended latent period, a slower average rate of growth and a less uniform pattern of growth. These effects result from localized radiation damage to host tissues, are not attributable to residual damage to irradiated tumour cells, and are not markedly dose-dependent within the dose range 25--80 Gy. These results are consistent with the complete sterilization of host endothelial cells by doses of 25 or more. In marked contrast to the growth-slowing effect of irradiation, the treatment of host animals by previous injection of radiation-killed tumour cells led to a reduced latent period and a faster average rate of growth.


*Br. J. Cancer* (1980) **41**, 567

### PERTURBATION OF THE GROWTH KINETICS OF C3H MOUSE MAMMARY CARCINOMA BY IRRADIATION OF TUMOUR AND HOST AND BY ATTEMPTED PRE-IMMUNIZATION OF HOST

A. S. ABDELAAL, T. E. WHELDON* AND B. M. CLARKE

*From the Radiobiology Research Group, Glasgow Institute of Radiotherapeutics and Oncology, Belvidere Hospital, Glasgow*

Received 30 July 1979 Accepted 14 November 1979

**Summary.—The kinetics of development and subsequent growth of a C3H mouse mammary tumour after implantation of $10^6$ cells was quantified by observation and statistical analysis of latent period and growth rate for different categories of tumour. These comprised control tumours, tumours recurring after large single doses of X-rays alone and in combination with misonidazole, tumours developing from cells implanted both outside and within the sites of tumours previously cured by irradiation, tumours developing from cells implanted in heavily irradiated skin, and tumours developing from cells taken from tumours recurring after irradiation and re-implanted in untreated skin. The kinetics of development and growth of tumours in host animals previously treated by i.p. injection of killed tumour cells was also quantified.**

**The results confirm that tumour development and growth is significantly perturbed by irradiation of host tissues both before and after tumour transplantation, and that this perturbation involves an extended latent period, a slower average rate of growth and a less uniform pattern of growth. These effects result from localized radiation damage to host tissues, are not attributable to residual damage to irradiated tumour cells, and are not markedly dose-dependent within the dose range 25–80 Gy. These results are consistent with the complete sterilization of host endothelial cells by doses of 25 or more.**

**In marked contrast to the growth-slowing effect of irradiation, the treatment of host animals by previous injection of radiation-killed tumour cells led to a reduced latent period and a faster average rate of growth.**

THE MODIFICATION of growth kinetics which occurs for tumours recurring after sub-curative therapy with large doses of radiation has been recognized for many years (Stenstrom *et al.*, 1955; Summers *et al.*, 1964; Thomlinson & Craddock, 1967). Despite this widespread recognition, quantitation of these effects in statistically valid terms remains insufficient, and has led to disagreement as to whether tumours do (Suit & Shalek, 1963; Breur, 1966) or do not resume their former growth rate, and whether there exist secondary modifications of growth as recurrent tumours approach their original size (Brown & Howes, 1974; McNally, 1974).

Such information is essential for any meaningful attempt to assess levels of tumour-cell survival by back-extrapolation of growth curves after irradiation (Hawkes *et al.*, 1968) or cytotoxic drugs (Berenbaum, 1972; Lloyd, 1975) or by methods of assay which require this assumption (Alfieri & Hahn, 1978).

In this study we seek to derive conclusions on the perturbation of tumour growth by X-rays alone and in combination with misonidazole (MISO). We have

* Present address: MRC Cyclotron Unit, Hammersmith Hospital, London W12.

also examined the possibility that tumour growth may be perturbed by previous exposure of host animals to killed tumour cells, a possibility of interest in relation to immunotherapy.

We have quantitatively examined the statistical distributions of latent period and/or growth rate for tumours belonging to the following experimental categories:

(a) Control tumours, *viz.* tumours developing from untreated cells implanted in untreated hosts;
(b) Tumours recurrent after sub-curative therapy with large single doses of X-rays alone and with MISO;
(c) Tumours developing from untreated cells implanted in heavily irradiated skin;
(d) Tumours developing from untreated cells implanted in the sites of tumours previously cured by X-rays alone or in combination with MISO;
(e) Tumours developing from untreated cells implanted just outside the sites of tumours previously cured by X-rays alone or in combination with MISO;
(f) Tumours developing from cells taken from tumours, in other hosts, recurrent after sub-curative therapy and re-implanted in untreated skin;
(g) Tumours developing from untreated cells implanted in hosts previously exposed to killed tumour cells by 3 inoculations, each of $2 \times 10^6$ radiation-sterilized cells in each of 3 preceding weeks.

### MATERIALS AND METHODS

*Tumour system.*—The multiple-generation C3H mouse mammary carcinoma is a poorly differentiated adenocarcinoma of spontaneous origin which has been routinely transplanted every 2–3 weeks since 1973 in syngeneic recipients. The histology, pattern of growth and gross response to irradiation are now stable. The rate of growth is very fast with a volume-doubling time of ~1 day at 1–2 mm diameter, lengthening to 3 days at 10–12 mm diameter.

The $TCD_{50}$ for X-rays is ~67 Gy. The "take rate" for $10^6$ cells implanted in the dorsal skin of untreated mice is close to 100%. No spontaneous regressions of well-established tumours have been recorded.

*Tumour transplantation technique.*—The tumour-cell suspension was prepared from a tumour of 10 mm diameter by gentle mashing, passage through a steel mesh and a series of needles of decreasing size, counting the number of cells judged as viable under a phase-contrast microscope, diluting to $10^6$ cells in 0·05 ml and implantation in the dorsum.

*Irradiation techniques.*—The X-irradiations were performed using a Siemens Stabiliplan machine operating at 250 kV and a filament current of 15 mA. Beam filtration was such as to give an experimentally determined first HVL of $1{\cdot}85 \pm 0{\cdot}05$ Cu and a dose rate of 1·10 Gy/min at 57 cm FSD.

All *in vivo* irradiations were carried out at room temperature, with mice breathing air and without anaesthesia in specially designed cylindrical jigs made of lead 2 mm thick to shield all the body of the mouse except the tumour. Extra lead sheets were used to limit the width of the field of irradiation to 1·5 cm, across which 4 tumours could be irradiated simultaneously in a tangential position. Since the X-ray beam was vertical, the mice were lying on their sides throughout the irradiation, a position which they tolerated well.

For "immunization" studies, a tumour-cell suspension of $10^6$ cells/0·05 ml was prepared as for routine transplantation, and irradiated in a flask with a single dose of 90 Gy at a dose rate of 1·28 Gy/min.

For experiments involving irradiation of cells to be implanted in unirradiated skin, the procedure was as for the "immunization" experiments, but with a single dose of only 70 Gy.

*Measurement of tumour size.*—Mice implanted with tumour cells were examined at intervals of 1–2 days, and the day of definite appearance of a palpable tumour (1–2 mm in diameter) was recorded. Subsequent growth was followed at daily intervals by measurement of 3 mutually perpendicular diameters using a specially-designed fan-shaped device fitted with a series of slits of graded size, to which each diameter could be compared, correct to the nearest millimeter. An average of 1·25 mm double skin thickness was obtained for these mice, and this value was subtracted from the average diameter measured for each tumour.

*Studies using misonidazole.*—These experiments made use of the hypoxic cell radiosensitizer MISO (kindly supplied by Professor G. E. Adams) made up as a sterile solution in 0·9% w/v saline at a concentration of 25 mg/ml and injected i.p. at a dose of 1 mg/g body wt 30 min before irradiation.

*Attempted immunization.*—For these experiments each mouse received 3 i.p. injections, each of $2 \times 10^6$ irradiated cells (0·1 ml suspension) at weekly intervals. The mice were challenged 3 weeks from the first injection with a tumour transplant, routinely prepared.

### EXPERIMENTS AND RESULTS

### *Gross response and curability of tumours treated with large single doses of X-rays with and without MISO*

The gross response to treatment, and rate of cure, of C3H mammary tumours have been described in detail elsewhere (Abdelaal & Nias, 1979) and are summarized here to provide background to the effect of radiation on this tumour system. These tumours showed a gross response pattern which was clearly dose-dependent for doses up to 50 Gy of X-rays alone (Fig. 1), or up to 25 Gy in the case of X-rays + MISO (Fig. 2) but was less obviously dose-dependent thereafter, the time-scale of complete visible regression for tumours exposed to these higher doses being ~ 3 weeks.

The tumour is not readily cured by low doses of radiation. No cures were seen for doses below 50 Gy for X-rays alone or below 25 Gy for X-rays + MISO. The curability of the tumour as a function of X-ray dose is recorded in Table I for X-rays alone and in Table II for X-rays + MISO.

The biological effectiveness of the doses used in the experiments described below may be assessed by comparison with Figs. 1 and 2, and with Tables 1 and II.

### *Distributions of latent period for the different experimental categories*

In these experiments, mice implanted with $10^6$ cells were inspected daily or near-daily and the number of days from implantation to first observation of a palp-

FIG. 1.—Gross tumour response to single dose of X-rays.

FIG. 2.—Gross tumour response to X-rays+MISO.

TABLE I.—*Curability of C3H mouse mammary carcinoma by single doses of X-rays alone*

| Dose (Gy) | Curability* (%) |
|---|---|
| ⩽50 | 0 |
| 60 | 11 |
| 65 | 33 |
| 70 | 66 |
| 75 | 82 |
| ⩾80 | 100 |

* Local control at 100 days. Since very few recurrences occur after 100 days, this is virtually synonymous with cure.

A total of 199 mice were used in these experiments. $TCD_{50}$ = 67 Gy.

TABLE II.—*Curability of C3H mouse mammary carcinoma by single doses of X-rays + misonidazole*

| Dose (Gy) | Curability (%) |
|---|---|
| ⩽20 | 0 |
| 25 | 5·8 |
| 27·5 | 6·2 |
| 30 | 61·5 |
| 35 | 75 |
| ⩾37·5 | 100 |

A total of 199 mice were used in these experiments. $TCD_{50}$ = 31 Gy, giving an apparent dose-modifying factor of 2·16 for MISO.

able tumour (typically 1–2 mm in diameter) which subsequently grew progressively was recorded for each individual mouse. These observations yielded distributions of latent period, the mean and standard deviation of which are recorded in Table III, for each of 5 experimental categories.

Statistical comparison of the significance of the difference of the means of each distribution from the control distribution was carried out using the rank-sum test (see Colquhoun, 1971) which has the advantage that it is not necessary to assume normality of the statistical distributions.

As may be seen from Table III neither tumours developing from cells implanted in previously cured sites, nor those from cells taken from tumours recurrent after subcurative therapy (*i.e.* "irradiated cells implanted in unirradiated skin") were significantly different in their mean latency from routine transplants in control mice. However, cells implanted in irradiated skin developed significantly more slowly,

TABLE III.—*Latent period for different experimental categories*

| Category | No. mice | Latent Period (days) Mean | s.d. | *P* for difference of mean from control |
|---|---|---|---|---|
| Control | 20 | 9·3 | 3·48 | — |
| Irradiated cells implanted in unirradiated skin (70 Gy) | 6 | 9·2 | 2·47 | >0·05 |
| Unirradiated cells implanted in irradiated skin (70 Gy) | 20 | 14·4 | 4·66 | <0·01 |
| Unirradiated cells implanted in sites of previously cured tumours (60–80 Gy X-rays only) | 20 | 8·4 | 3·10 | >0·05 |
| Unirradiated cells implanted in "Immunized" hosts | 20 | 4·85 | 2·25 | <0·01 |

Mice implanted with $10^6$ viable cells developed tumours with 100% frequency in all categories *except* that of "irradiated cells implanted in unirradiated skin". In this category, 10 mice were implanted but only 6 tumours developed.

and cells implanted in "immunized" hosts developed significantly more rapidly, than controls.

*Rate of growth of tumours in the first* 10 *days after detection*

The rates of growth of tumours in the 10 days immediately after the first definite detection (typically at 1–2 mm in diameter) was measured for tumours belonging to 6 different experimental categories. Specifically excluded from this analysis, however, are the categories of tumours recurrent after therapy with X-rays alone or in combination with MISO. The pattern and rate of growth of recurrent tumours is considered in the following section.

However, for the great majority of tumours in the 6 categories considered here, it was convenient to quantify growth in terms of the linear increase of diameter with time, using the method of least squares to determine best linear slopes. The growth curve of each tumour was recorded, and the analysis was carried out for each tumour individually.

An advantage of analysis of growth curves of individual tumours is that it facilitates the detection of the occasional "rogue" tumour the growth pattern of which sets it apart from the others in the same category.

Thus, although most tumours followed a linear pattern of increase of diameter with time (with correlation coefficients typically in the region of 0·95–0·99) a small minority of tumours followed a more erratic pattern (similar to that seen with most of the recurrent tumours) and were excluded from this analysis on the arbitrary basis of a correlation coefficient

TABLE IV.—*Rate of tumour growth for* 10 *days after detection*

| Tumour group | No. evaluated | Growth rate (mm/day) Mean | s.d. | *P* for difference of mean growth rate from control |
|---|---|---|---|---|
| Control | 31 | 0·889 | 0·164 | — |
| Cells implanted outside cured sites | 11 | 0·827 | 0·170 | >0·05 |
| Cells implanted at site of tumours cured with X-rays only (60–80 Gy) | 16 | 0·493 | 0·170 | <0·001 |
| Cells implanted at site of tumours cured with X-rays (25–50 Gy)+MISO | 16 | 0·435 | 0·170 | <0·001 |
| Cells taken from tumours recurrent after X-irradiation (50–60 Gy) and implanted in untreated skin | 6 | 1·175 | 0·149 | >0·05 |
| Cells implanted in irradiated skin (70 Gy) | 14 | 0·571 | 0·122 | <0·001 |
| Cells implanted in "immunized" hosts | 20 | 1·06 | 0·282 | <0·05 |

under 0·90. (In fact, most included tumours had coefficients greater than 0·95 and most excluded tumours had coefficients less than 0·75.) The significance of the excluded minority will be considered in subsequent sections.

For the great majority of tumours included in the analysis, the results are shown in Table IV, from which it may be seen that neither cells implanted beyond cured sites, nor previously irradiated cells taken from recurrent tumours, gave rise to tumours whose rate of growth differed significantly from those of control tumours. Cells implanted in previously cured sites and in previously irradiated skin grew significantly more slowly than controls. It is of interest that cells implanted in sites of tumours cured by X-rays alone or by X-rays plus MISO gave rise to tumours having rather similar growth rates, despite the disparity in X-ray doses (60–80 Gy and 25–50 Gy respectively).

By complete contrast, tumours developing from cells implanted in "immunized" hosts grew significantly *faster* than did controls.

*Rate of growth of tumours in the* 10 *days before animal killing*

For unirradiated tumours which grew reasonably quickly (*i.e.* ~1 mm/day) 10 days from initial detectability at a diameter of 1–2 mm produced tumours with mean diameter > 10 mm, after which it became increasingly likely that animal killing would be required on humane grounds.

However, for tumours which grew more slowly, longer periods of observation were feasible, allowing comparison of growth in the "early" and "late" phases of growth after detection. A useful definition of such phases is "early" for the phase 10 days after detection and "late" for the phase 10 days before killing. For rapidly growing tumours these phases were virtually the same, but for slowly growing tumours they were overlapping but not identical.

In addition, it was difficult to determine the growth rates of recurrent tumours in the "early" phase, which usually grew in a highly erratic fashion, with periods of slow growth interspersed with periods in which transient secondary regressions took place. The growth of these tumours, excluded from consideration in the previous section, is most readily quantified for the "late" phase only.

Tumour growth in the "late" phase is tabulated in Table V. Comparison of Tables IV and V shows that, for irradiated tumours the growth of which was quantified in both the "early" and "late" phases, there was a tendency for growth to accelerate from the "early" to the "late" phase though, in general, growth remained slower than that of unirradiated tumours.

The growth retardation of recurrent tumours during the "early" growth phase was not readily assessed by linear analysis, but some idea of the magnitude of the effect may be obtained from Table VI which shows the mean time taken to grow from 3 mm to 10 mm diameter for

TABLE V.—*Rate of tumour growth for* 10 *days before killing*

| Tumour group | No. evaluated | Growth rate (mm/days) Mean rate | s.d. | *P* for difference of mean growth rate from control |
|---|---|---|---|---|
| Control | 31 | 0·889 | 0·164 | — |
| Cells implanted in sites of tumours previously cured with X-rays (60–80 Gy) | 16 | 0·81 | 0·182 | > 0·05 |
| Cells implanted in irradiated skin (70 Gy) | 14 | 0·73 | 0·192 | < 0·01 |
| Tumours recurrent after 50 Gy X-rays | 8 | 0·79 | 0·109 | > 0·05 |
| Tumours recurrent after 60 Gy X-ray dose | 8 | 0·64 | 0·134 | < 0·01 |
| Tumours recurrent after 65 Gy X-ray | 7 | 0·71 | 0·148 | < 0·01 |

TABLE VI.—*Times for tumours to grow from a mean diameter of 3 mm to one of 10 mm*

| Tumour group | No. of tumours | Mean (days) | S.d. |
|---|---|---|---|
| Control | 50 | 8·7 | 0·22 |
| Tumours recurrent after X-rays only (> 60 Gy) | 19 | 28·4 | 2·2 |
| Tumours recurrent after X-rays + MISO (> 25 Gy) | 10 | 24·9 | 3·1 |
| Cells implanted at sites of tumours previously cured by X-rays only (> 60 Gy) | 33 | 14·3 | 0·64 |
| Cells implanted at sites of tumours previously cured by X-ray + MISO (> 25 Gy) | 19 | 14·6 | 0·88 |
| Cells implanted in irradiated skin (70 Gy) | 14 | 13·9 | 0·54 |

tumours in different categories. The striking feature of this table is the time taken for recurrent tumours, compared to tumours developing from cells implanted in cured sites or in irradiated skin.

Since, as seen from Table V, the "late" phase growth rates of these tumours were not markedly different, the differences seen in Table VI provide a measure of the "early", non-linear growth retardation to which recurrent tumours seemed especially prone.

*Uniformity of patterns of tumour growth*

As stated in the previous sections, only in the case of the recurrent tumours did the pattern of tumour growth conform poorly to the model of linear increase of diameter with time, though small numbers of "rogue" tumours with similarly erratic behaviour also occurred as a minority group in some other categories.

In an attempt to quantify this phenomenon, Table VII shows the numbers, and statistical significance for tumours excluded from growth-rate analysis. The results show that such "rogue" tumours are confined to tumours growing in sites previously subjected to irradiation, and that their frequency in sites of previously cured tumours is statistically significant.

TABLE VII.—*Uniformity of tumour growth for 10 days after detection*

| Tumour group | No. tumours | No. excluded on grounds of nonlinear growth kinetics | *P* for No. exclusions relative to control |
|---|---|---|---|
| Control | 31 | 0 | — |
| Cells implanted in sites of tumours cured by X-rays alone (50–80 Gy) | 20 | 4 | < 0·05 |
| Cells implanted in sites of tumours cured by X-rays + misonidazole (25–50 Gy) | 20 | 4 | < 0·05 |
| Cells implanted in irradiated skin (70 Gy) | 14 | 2 | > 0·05 |
| Cells implanted outside the sites of previously cured tumours | 11 | 0 | — |
| Cells implanted in "immunized" hosts | 20 | 0 | — |

Inspection of the data on the growth of such tumours shows a growth rate which is quite low, but consists of sub-phases of active growth interrupted by phases of no growth or even transient regression.

Taken together with the observations on recurrent tumours, the data of Table VII indicate that irradiation decreases the uniformity as well as the rate of subsequent tumour growth. It is, however, of interest that "rogue" tumours formed most of the recurrent tumours (in the "early" growth phases) but were a small minority in other irradiated groups.

### DISCUSSION AND CONCLUSIONS

The studies reported here are in broad agreement with those of other workers. However, we have extended the observations to a higher range of X-ray doses, to treatments employing MISO in combination with X-rays and to the effect of prior "immunization" of host on the kinetics of development and growth of mouse mammary tumours.

In addition we have monitored and analysed the growth curve for each tumour individually, a procedure useful for the identification of occasional "rogue" tumours the kinetic behaviour of which makes them untypical of their experimental group.

*Effect of irradiation on the latency and average rate of tumour growth*

For the great majority of tumours the uniformity of growth of which lends itself to linear analysis, the observation of normal latency and normal growth rates in tumours developing from cells of tumours recurrent after irradiation and implanted in unirradiated skin, but of prolonged latency and slower growth in tumours developing from unirradiated cells implanted in irradiated skin, confirms that normal tissue damage, rather than residual damage to tumour cells, is primarily responsible for the perturbation of development and growth of tumours irradiated *in vivo*. This conclusion is also supported by the at least partial recovery of growth rate as the tumour extends beyond the original irradiated area.

It is of interest that the perturbation which results from irradiation of host tissues causes a prolonged latent period, as well as a reduced growth rate, a finding also reported by Urano & Suit (1971), though not by Hewitt & Blake (1968). This suggests that the perturbation of growth could apply to microscopic as well as macroscopic tumours growing in heavily irradiated environments, though studies with different numbers of implanted cells would be required to confirm this.

The magnitude of the effect on growth rate during the "early" phase, an approximate halving of the linear rate of increase of diameter with time for X-ray doses in the range 60–80 Gy and, when in combination with the hypoxic sensitizer MISO for doses in the range 25–50 Gy, is similar to that reported by Hewitt & Blake (1968) for the dose range 10–40 Gy.

The similarity of perturbation caused by 60–80 Gy X-rays alone or 25–50 Gy of X-rays + MISO suggests either that normal tissues are appreciably hypoxic or that the growth perturbation is not markedly dependent on X-ray dose above a threshold which is probably less than 25 Gy. The latter interpretation is consistent with those of other studies (Summers *et al.*, 1964; Urano, 1966; Hewitt & Blake, 1968) and with our own failure to detect a monotonic relationship between mean growth rate and dose for recurrent tumours.

This implies a "plateauing" of the effect over a very large dose range ($<25$ Gy–$>80$ Gy) a phenomenon not readily explained in terms of the radiation killing of endothelial stem cells. If recurrent tumours succeed in evoking a blood supply from host tissues irradiated to doses as high as 80 Gy, the endothelial cells responsible for capillary formation must (if not hypoxic) be extraordinarily resistant to X-rays, or, conceivably, migrate in to the irradiated volume from surrounding tissues or from blood (Hewitt & Blake, 1968; Urano & Suit, 1971).

The differences between the results at the cured and pre-irradiated sites would suggest structural and/or functional differences at these sites. These might be the different radiation responses of vascularity already existing at the time of tumour irradiation and of non-stimulated vascularity at the pre-irradiated sites.

A growth similar to that of recurrent tumours might have been obtained at the cured sites if fewer tumour cells (less than $10^6$) had been used for transplantation. This would need to be confirmed by a dilution assay, using various concentrations of tumour-cell suspension for transplantation.

Recurrent tumours, and those developing at the sites of previously cured tumours, may be able to use a pre-existing structurally intact vascular system (whatever the clonogenic status of constituent cells) but cells implanted in irradiated tissue might be expected to be at a relative disadvantage in this case.

This could perhaps explain the relatively prolonged latency (Table III) of tumours developing in irradiated tissue though, once tumour development had taken place, the rate of subsequent growth was not dissimilar to that of tumours developing in previously cured sites.

In total, these studies reinforce the concept that the perturbation of tumour growth seen after irradiation is primarily due to normal tissue injury, particularly of the vascular elements, and so constitutes a "tumour bed effect". Thrombosis of vessels and a general diminution of the blood supply available to the tumour seem plausible mechanisms for the effect (Thomlinson & Craddock, 1967; Clifton & Jirtle, 1975; Jirtle *et al.*, 1978).

In this case, it is probable that not only the rate and uniformity of growth, but also the oxygenation status of tumours growing in irradiated tissues, will be perturbed. If, as indicated here, the effect prolongs latency also, the meaningful interpretation of tumour regrowth delay in terms of cell survival cannot be undertaken without knowledge of quantitative aspects of the phenomenon.

*Effect of irradiation on uniformity of tumour growth*

The irregularity of "early" growth of recurrent tumours, a feature long recognized but seldom quantified, presents difficulties to meaningful analysis of recurrence and regrowth delay. Though regrowth delays are often taken as the time for the tumour to grow to *greater* than its pre-irradiated size (in effect, to allow it to reach the "late" growth phase), the difficulty is not wholly avoided if an erratic and highly variable phase of growth contributes to the recorded delay.

If non-uniformity is imposed by vascular damage to host tissues, it is not apparent why "rogue" tumours should be the norm for the recurrent tumours but only a minority for other irradiated sites. One possibility is that the diminished frequency of "rogues" in the irradiated skin and cured-site categories is a consequence of the time of appearance (subsequent to irradiation) of tumours in these different groups. Thus recurrent tumours always appeared within 100 days of irradiation, whereas cells were implanted in irradiated skin 100–110 days after irradiation, and in cured sites 100–250 days after irradiation.

However, there have been, to our knowledge, no attempts to quantitatively assess the perturbation of uniformity of growth, a feature no less important than rate of growth to the understanding of *in vivo* tumour responses. It is possible that, using the form of analysis described here, this could be done in the future.

*Kinetic effects of "pre-immunization"*

One of the most intriguing observations has been the effect of "pre-immunization" on the kinetics of development and growth of a subsequently transplanted tumour. Not only did "pre-immunization" fail to prevent transplantation or retard development or growth, but all such transplanted tumours developed and grew significantly faster than the non-immunized controls.

It is difficult to say whether the effect is immunologically mediated. Certainly, immunological effects cannot be excluded, in view of the possibility of the immune response acting as a growth-promoting mechanism (Prehn, 1972). It is also of interest that the presence of the tumour seems to depress the immunocompetence of the host, at least as measured by the ability of host lymphocytes to respond to the mitogen PHA *in vitro*—an effect abolished by cure of the tumour (Abdelaal *et al.*, 1978).

Whatever the mechanisms involved, the results presented here provide further evidence that, at least in some tumour systems, inoculation with killed tumour cells may lead to faster development and faster subsequent growth of transplanted tumours.

REFERENCES

ABDELAAL, A. S., CHAMBERLAIN, S. M., NIAS, A. H. W. & WALKER, A. (1978) Metastases and immunological studies in C3H mice after hyperthermia. *Br. J. Radiol.*, **51**, 937.

ABDELAAL, A. S. & NIAS, A. H. W. (1979) Regression, recurrence and cure in an irradiated mouse tumour. *J. R. Soc. Med.*, **72**, 100.

ALFIERI, A. & HAHN, E. W. (1978) An *in situ* method for estimating cell survival in a solid tumour. *Cancer Res.*, **38**, 3006.

BERENBAUM, M. C. (1972) *In vivo* determination of the fractional cell kill of human tumour cells by chemotherapeutic agents. *Cancer Chemother. Reps.*, **56**, 563.

BREUR, K. R. (1966) Growth rate and radiosensitivity of human tumours. *Eur. J. Cancer*, **2**, 157.

BROWN, J. M. & HOWES, A. E. (1974) Comparison of tumour growth delay with cell survival. *Br. J. Radiol.*, **47**, 509.

CLIFTON, K. H. & JIRTLE, R. (1975) Mammary carcinoma population growth in pre-irradiated and unirradiated transplant sites. *Radiology*, **117**, 459.

COLQUHOUN, D. (1971) *Lectures on Biostatistics.* Oxford: Clarendon Press.

HAWKES, M. J., HILL, R. P., LINDOP, P. J., ELLIS, R. E. & ROTBLAT, J. (1968) The response of C3H mammary tumours to irradiation in single and fractionated doses. *Br. J. Radiol.*, **41**, 134.

HEWITT, H. B. & BLAKE, E. R. (1968) The growth of transplanted tumours in pre-irradiated sites. *Br. J. Cancer*, **22**, 808.

JIRTLE, R., RANKIN, J. H. G. & CLIFTON, K. H. (1978) Effects of X-irradiation of tumour bed on tumour blood flow and vascular response to drugs. *Br. J. Cancer*, **37**, 1033.

LLOYD, H. R. (1975) Estimation of tumour cell kill from Gompertz growth curves. *Cancer Chemother. Rep.*, **59**, 267.

MCNALLY, N. M. (1974) Tumour growth and cell survival *in situ*. *Br. J. Radiol.*, **47**, 510.

PREHN, R. T. (1972) The immune reaction as a stimulator of tumour growth. *Science*, **176**, 170.

STENSTROM, K. W., VERMUND, H., MOSSER, D. G. & MARVIN, J. F. (1955) Effects of röntgen radiation on the tumour bed. *Radiat. Res.*, **2**, 180.

SUIT, H. D. & SHAKEK, R. J. (1963) Response of anoxic C3H mouse mammary carcinoma isotransplants to X-irradiation. *J. Natl Cancer Inst.*, **31**, 479.

SUMMERS, W. C., KLIFTON, K. H. & VERMUND, H. (1964) X-irradiation of the tumour bed. I: A study of the indirect actions of radiation on transplantable tumours. *Radiology*, **82**, 691.

THOMLINSON, R. H. & CRADDOCK, E. A. (1967) The gross response of an experimental tumour to single doses of X-rays. *Br. J. Cancer*, **21**, 108.

URANO, M. (1966) Effect of X-irradiated tumour bed on tumour cells. I: Effect on tumour growth and host survival. *Nippon Acta Radiol.*, **26**, 1372.

URANO, M. & SUIT, H. D. (1971) Experimental evaluation of tumour bed effect of C3H mouse mammary carcinoma and for C3H mouse fibrosarcoma. *Radiat. Res.*, **45**, 41.